# Climate-Driven Redistribution of Early-Spring Ephemeral Plant Communities in Cold Arid Deserts: Evidence from the Gurbantunggut Desert, China

**DOI:** 10.3390/plants15101586

**Published:** 2026-05-21

**Authors:** Yang Xue, Jiazheng Ma, Songmei Ma, Yuting Chen, Xu Sun, Mengyuan Ren, Liqiang Shen

**Affiliations:** College of Urban and Environmental Sciences, Shihezi University, Shihezi 832003, China; xueyang@stu.shzu.edu.cn (Y.X.);

**Keywords:** early-spring ephemeral plants, future climate change, remote sensing extraction, spatiotemporal distribution patterns, machine learning

## Abstract

Early-spring ephemeral plants act as pioneer species on stabilized dunes in cold arid deserts; they are capable of rapid growth under extreme drought and low-temperature conditions while sustaining dune ecosystem functions. These species are highly sensitive to climate change, yet their spatiotemporal dynamics and the mechanisms by which climatic factors regulate their growth remain poorly understood. In this study, we investigated the Gurbantunggut Desert, China, using long-term NDVI time series to extract phenological traits associated with their life cycle and developed a remote-sensing-based analytical framework to quantify the distribution patterns of early-spring ephemeral plants and their environmental drivers. We combined random forest (RF), structural equation modeling (SEM), and convolutional neural networks (CNN) to assess the relative importance and pathways of key climatic drivers and to predict future distribution changes. Our results indicate that: (1) the life cycle extraction method achieved a classification accuracy exceeding 80%, and from 2001 to 2022, the overall distribution of early-spring ephemeral plants exhibited an increasing trend; (2) snowend, snowday, and precipitation during the driest quarter were the primary drivers of ephemeral plant distribution, collectively explaining over 60% of the observed variation, and structural equation modeling further revealed that snow and precipitation had significant positive effects on their distribution; and (3) under future climate scenarios, Medium-NDVI areas are projected to expand northward and westward, with the potential emergence of new suitable habitats in northern localities by mid-century. Climate warming may facilitate the dispersal and latitudinal migration of early-spring ephemeral plants. Based on these findings, biodiversity conservation efforts should prioritize ecologically sensitive transitional zones and promote species migration and establishment under climate change through the construction of ecological corridors.

## 1. Introduction

According to the Fifth Assessment Report of the Intergovernmental Panel on Climate Change (IPCC), the global warming trend is projected to continue [1]. By the end of this century, the global mean temperature is expected to increase by 0.3 to 4.8 °C [2,3]. Climate change characterized by global warming may cause habitat loss or fragmentation, posing substantial threats to species survival and distribution [4,5]. Particularly in arid and semi-arid regions, rising temperatures and altered precipitation patterns are likely to exacerbate soil moisture deficits and habitat fragmentation, thereby profoundly affecting plant community structure and species diversity [6]. Owing to their distinctive continental climate characteristics, the arid and desert ecosystems in northwestern China are extremely fragile and highly sensitive to climate change. The structure, function, and stability of these ecosystems have already been severely disrupted, compromising oasis ecological security and sustainable agricultural development [7,8,9]. As a vital component of arid ecosystems, desert vegetation plays a critical role in stabilizing sand dunes and combating desertification [10]. Therefore, investigating the dynamic changes of desert vegetation and the underlying climatic driving mechanisms is essential for understanding how regional ecosystems respond to climate change and for formulating appropriate ecological conservation and restoration strategies.

Desert early-spring ephemeral plants represent a distinctive group of pioneer herbaceous communities in cold arid deserts, primarily distributed across Central Asia and northwestern China. They can quickly complete the whole life cycle from seed germination to seed death in 2–3 months by using snow melt water and precipitation in early spring [11,12]. Despite their short growth period, early-spring ephemeral plants play an irreplaceable and critical role in cold arid desert ecosystems. As pioneer species in sand dune stabilization, their coverage can exceed 40% during the peak wind–sand activity period in May, effectively reducing near-surface wind speed, mitigating wind erosion, and maintaining dune stability [13]. In addition, the decomposition of their litter provides essential matter and energy inputs to desert ecosystems, promotes nutrient cycling and soil amelioration, and enhances the stability of desert ecosystems [14,15,16]. As the second-largest desert in China, the Gurbantunggut Desert is home to about 66 species of ephemeral plants, accounting for 32.5% of the total ephemeral plants in northern Xinjiang and 31.7% of the total desert plant species [17]. It is crucial to maintain the function of desert ecosystems.

Existing studies on desert early-spring ephemeral plants have mainly focused on two aspects. The first is physiological research at the individual and population levels, centering on seed germination characteristics, flowering, and responses to microenvironmental changes such as water and temperature conditions. For instance, Chen et al. [18] investigated the responses of seeds of 28 ephemeral plant species to temperature and light, as well as their germination speed; Zeng et al. [13] revealed the flowering regulatory network of *Eremopyrum triticeum* at the genetic level; and Wang et al. [19] demonstrated that the germination and growth of ephemeral plants are sensitive to changes in hydrothermal conditions. The second aspect concerns ground-based surveys at the community level, primarily focusing on species diversity, community structure, and small-scale spatial distribution patterns. Related studies have systematically investigated the floristic composition of early-spring ephemeral plants in northern Xinjiang, documenting 205 ephemeral plant species [12]. However, owing to their broad distribution ranges, short life cycles, and strong spatial heterogeneity, traditional field survey methods cannot effectively monitor their dynamics at large spatial scales and over long time series. Moreover, existing studies on driving mechanisms have predominantly concentrated on changes in water and temperature, lacking the quantification of the multi-factor comprehensive action path.

In recent years, the development of remote sensing technology has provided an important tool for monitoring vegetation dynamics at the regional scale, offering new opportunities to investigate the spatiotemporal dynamics of desert early-spring ephemeral plants [20,21]. The Normalized Difference Vegetation Index (NDVI), as one of the most widely used vegetation indices, can effectively reflect vegetation growth conditions and seasonal variation characteristics, and has been extensively applied in studies of vegetation cover change, phenology monitoring, and ecosystem responses to climate change [22,23]. At the methodological level, machine learning, owing to its strong nonlinear fitting capacity, has been increasingly used in recent years for analyzing driving mechanisms of ecological processes and predicting species distributions. For example, the Random Forest (RF) model can effectively identify the key drivers influencing ecological processes [24], while the Convolutional Neural Network (CNN) model, with its superior spatial feature learning ability, achieves high accuracy in long-term simulations and predictions [25,26]. Meanwhile, in future climate change research, the Shared Socioeconomic Pathway (SSP) scenarios proposed based on CMIP6 provide an important data foundation for assessing potential ecosystem changes under different climate change scenarios [27]. However, studies that integrate remote sensing time series analysis, machine learning methods, and future climate scenario projections to systematically investigate the spatiotemporal distribution and driving mechanisms of desert early-spring ephemeral plants remain scarce, making it difficult to support desert ecosystem management in the context of climate change.

Based on this, this study takes the Gurbantunggut Desert in Xinjiang as the study area, identifies the distribution of desert early-spring ephemeral plants using high-temporal-resolution NDVI data and life cycle theory, and integrates multi-source data and machine learning methods to explore the potential driving mechanisms underlying their distribution. On this basis, we further predict the potential distribution changes of desert early-spring ephemeral plants under different future climate conditions based on SSP climate scenarios. Specifically, this study aims to: (1) diagnose and visualize the spatiotemporal distribution patterns of NDVI of early-spring ephemeral plants in the Gurbantunggut Desert from 2001 to 2022, and track the migration pathways of different NDVI categories; (2) reveal the key limiting environmental factors that influence their distribution at different temporal scales; (3) predict the potential distribution patterns of NDVI of desert early-spring ephemeral plants under different climate scenarios using a convolutional neural network (CNN) model. The findings will help deepen the understanding of vegetation dynamics and their climatic response mechanisms in arid regions, and provide a scientific basis for regional ecological conservation and desert ecosystem management.

## 2. Results

### 2.1. Remote Sensing Diagnosis Extraction of NDVI and Accuracy Assessment

**Figure 1:**
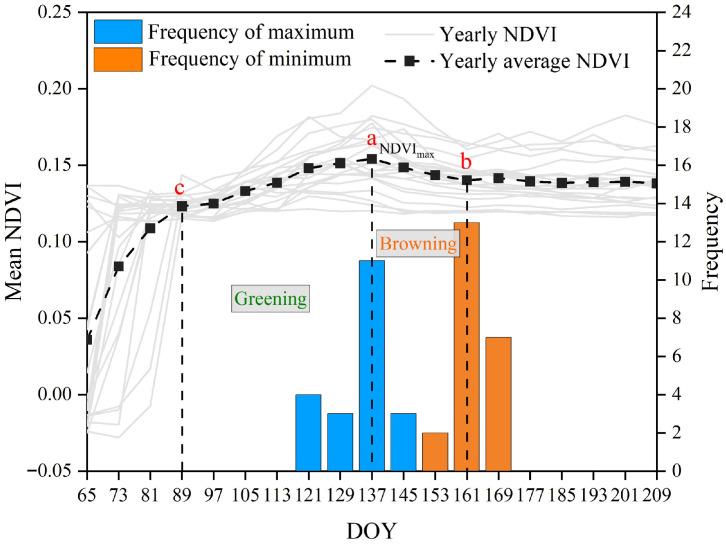
NDVI time series curve and extreme value frequency distribution of the maximum rectangular experimental area in the Gurbantunggut Desert (2001–2022). “a” represents the peak growth point; “b” represents the post-peak minimum point; “c” represents the regreening onset point.

The NDVI time series curves from 2001 to 2022 show that the NDVI of early-spring ephemeral plants peaked at Day 137 (17 May, point a), then declined rapidly and reached its minimum at Day 161 (18 June, point b) (Figure 1). The highest frequencies of extreme values occurred at Day 137 and Day 161. Validation based on actual species distribution data yielded an accuracy of 82.16% (Table 1), indicating that the extraction results are reliable.

### 2.2. Spatio-Temporal Characteristics Analysis

#### 2.2.1. Temporal Characteristics

**Figure 2:**
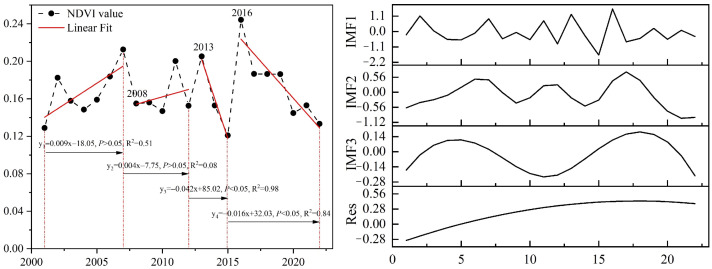
Temporal variation characteristics and periodic oscillation decomposition of NDVI for early-spring ephemeral plants within the study area (2001–2022).

The piecewise regression results indicate that the NDVI time series in the study area was divided into four segments: P1 (2001–2007), P2 (2008–2012), P3 (2013–2015), and P4 (2016–2022) (Figure 2). Among these, P1 and P2 exhibited a non-significant increasing trend, while P3 and P4 showed a significant decreasing trend. However, the mean NDVI values for 2013 and 2016 were relatively high, reaching 0.2053 and 0.2442, respectively. The EEMD model revealed that the NDVI of early-spring ephemeral plants in the study area exhibited periodic variations with periods of 1.4, 3.0, and 6.5 years. Furthermore, the residual component (Res) indicated a long-term, slow increasing trend (Figure 2). The variance contribution rate of Intrinsic Mode Function 1 (IMF1) was 67.22%, signifying strong short-term periodicity in NDVI, with the primary fluctuations dominated by the 1.4-year cycle (Table 2).

#### 2.2.2. Spatial Characteristics

**Figure 3:**
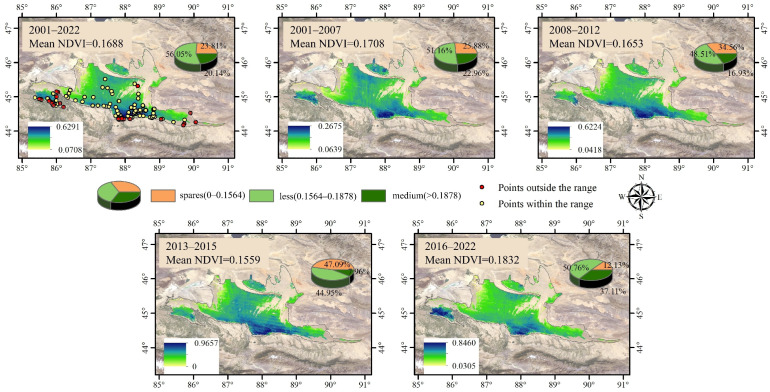
Accuracy validation of NDVI for early-spring ephemeral plants and classification extraction results in the Gurbantunggut Desert (2001–2022).

Spatially, the distribution of early-spring ephemeral plants was primarily concentrated in the southern margin and central region of the Gurbantunggut Desert (Figure 3). The spatial pattern exhibited higher NDVI values in the south than in the north, and higher values in the center than in the eastern and western regions. Approximately 50% of the ephemeral plant distribution fell within the Less-NDVI level, followed by the Sparse-NDVI level, with the Medium-NDVI level accounting for the lowest proportion.

**Figure 4:**
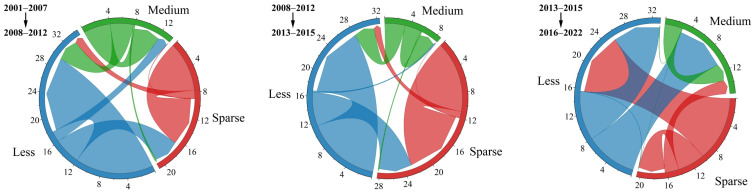
Transfer pathways of NDVI levels for early-spring ephemeral plants in the Gurbantunggut desert from 2001 to 2022 (Unit: 10^3^ km^2^).

From P1 to P2 and from P2 to P3, the NDVI level of early-spring ephemeral plants showed a decreasing trend (Figure 4 and Table 3). This was primarily manifested as a shift in area from the Medium-NDVI to the Less-NDVI category and from the Less-NDVI to the Sparse-NDVI category. The proportion of Medium-NDVI decreased from 22.96% to 7.96%. Conversely, during the P3 to P4 period, the NDVI level significantly improved, with the mean value increasing from 0.1559 to 0.1832. The area classified as Medium-NDVI increased by approximately 29.15%.

#### 2.2.3. Stability Analysis

**Figure 5:**
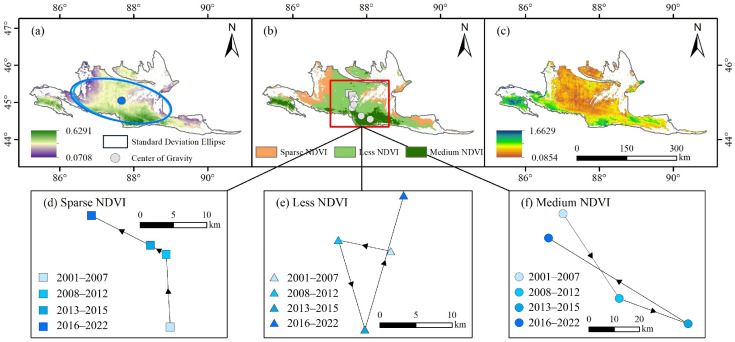
NDVI stability analysis (**a**,**c**) and centroid migration (**b**,**d**–**f**) of early-spring ephemeral plants (2001–2022).

The centroid shift results consistently indicated that the NDVI of early-spring ephemeral plants was relatively stable overall (Figure 5a). However, the centroids of the different NDVI categories exhibited significant movement from P1 to P4, all shifting towards higher latitudes (Figure 5b). Notably, the centroid of the Medium-NDVI category, which was primarily distributed in the southern margin of the desert, displayed the largest migration magnitude, shifting 51.99 km from P3 to P4. The CV results further revealed that NDVI fluctuations of early-spring ephemeral plants were more intense in the southern desert margin than in other regions (Figure 5c).

### 2.3. Driving Mechanism Analysis

#### 2.3.1. Relative Importance of Driving Factors

**Figure 6:**
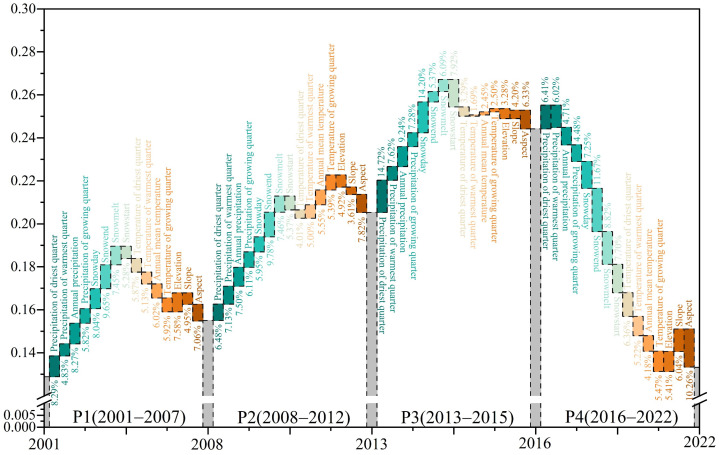
Contributions of climatic and topographic factors to NDVI variations of early-spring ephemeral plants in the Gurbantunggut Desert across the four periods (P1–P4). Gray bars (from left to right) represent the NDVI values in 2001, 2008, 2013, 2016, and 2022, respectively.

Across all four periods (P1–P4), the relative contribution of environmental factors to the NDVI of early-spring ephemeral plants ranked as follows: snow factors (32.49%) > precipitation factors (28.76%) > topographic factors (19.42%) > temperature factors (19.33%). The three most influential specific factors were Snowend, precipitation of the driest quarter (January–March), and Snowday (Figure 6).

Correlation analysis showed that snow and precipitation factors were positively correlated with NDVI, whereas temperature and topographic factors were predominantly negatively correlated (Supplementary Materials, Figure S1). During P1, a delayed snow end date and increased precipitation during the driest quarter significantly promoted higher NDVI. In P2, the influence of slope aspect became prominent, with higher NDVI values observed on north-facing slopes than on south-facing slopes. During P3, an increase in the number of snow days and precipitation during the driest quarter provided critical moisture conditions that supported ephemeral plant growth. In P4, the contribution of precipitation diminished, while the contribution of temperature increased significantly compared to P3. Slope aspect played a crucial role in reshaping the distribution pattern of early-spring ephemeral plants.

#### 2.3.2. Impact Pathway Analysis

**Figure 7:**
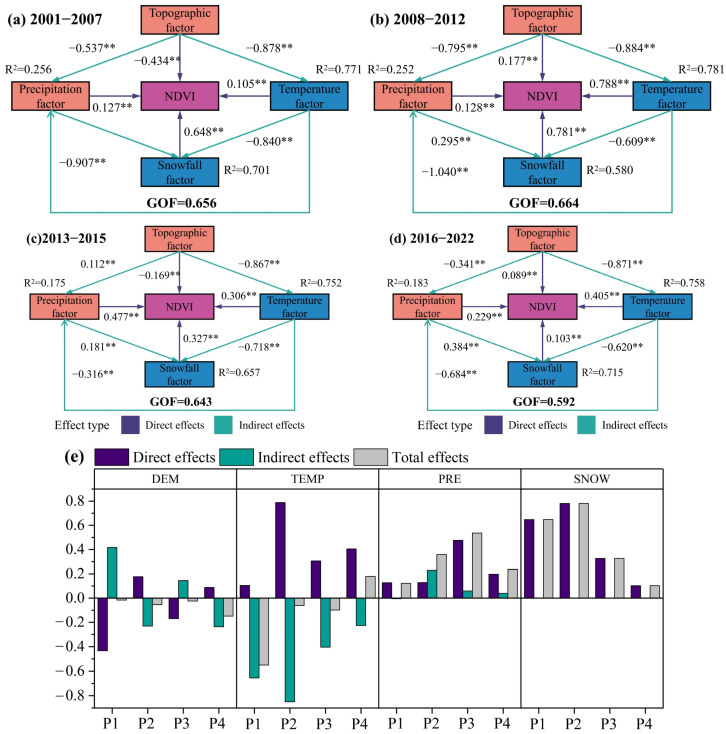
(**a**–**d**) SEM results for P1 (2001–2007), P2 (2008–2012), P3 (2013–2015), and P4 (2016–2022), respectively. Purple arrows: direct pathways; cyan arrows: indirect pathways. Path coefficients are shown with ** *p* < 0.01. (**e**) Total effects (direct + indirect) of snow, precipitation, temperature, and topography on NDVI across the four periods.

Across the P1–P4 periods, the snow factor and precipitation factor exerted an overall positive total effect on the NDVI of early-spring ephemeral plants, primarily through direct pathways. In contrast, the temperature factor and topographic factor exhibited an overall negative total effect, with the temperature factor acting mainly through indirect negative pathways (Figure 7).

In P1, on the direct pathway, the snow factor had the strongest positive impact on NDVI, with a path coefficient of 0.648. On the indirect pathway, the “Temperature → Precipitation” path showed the largest negative effect, with a coefficient of −0.907 (Figure 7a). In P2, on the direct pathway, the temperature factor and snow factor played dominant roles in driving NDVI changes, with coefficients of 0.788 and 0.781, respectively. On the indirect pathway, the “Topography → Temperature” path exerted the strongest influence on NDVI and was negative. Rising temperatures accelerated snowmelt, increasing water loss to the atmosphere and thereby negatively affecting the NDVI of early-spring plants (Figure 7b). In P3, on the direct pathway, the precipitation factor had the most pronounced influence, with a coefficient of 0.477. On the indirect pathway, negative effects mediated mainly by the topographic factor and temperature factor remained dominant (Figure 7c). In P4, the NDVI of early-spring ephemeral plants was primarily regulated by the temperature factor and precipitation factor, both of which exerted positive influences (Figure 7d).

### 2.4. Prediction of Early-Spring Ephemeral Plant NDVI Distribution in the Gurbantunggut Desert Under Future Climate Scenarios

#### 2.4.1. Screening of Key Driving Variables

Based on the ranking of driver contributions during P1–P4 and targeting a cumulative contribution exceeding 85%, this study selected 12 ecologically and geographically relevant environmental variables to construct a CNN model for predicting the spatial distribution of NDVI (Figure 6). These variables included: precipitation of the driest quarter, precipitation of the warmest quarter, March–June precipitation, annual precipitation, Snowday, Snowstart, Snowend, Snowmelt, temperature of the driest quarter, March–June temperature, elevation, and slope aspect. In constructing the future prediction model, future climate data were used for precipitation of the driest quarter, precipitation of the warmest quarter, annual precipitation, and temperature of the driest quarter, while the remaining variables were held constant.

#### 2.4.2. Simulation and Prediction of Spatio-Temporal Changes in Early-Spring Ephemeral Plant NDVI Based on the CNN Model

Five-fold cross-validation and comparison with actual observational data revealed that the model achieved satisfactory accuracy, with a coefficient of determination (R^2^) of 0.857 ± 0.019 and a root mean square error (RMSE) of 0.013 ± 0.001.

**Figure 8:**
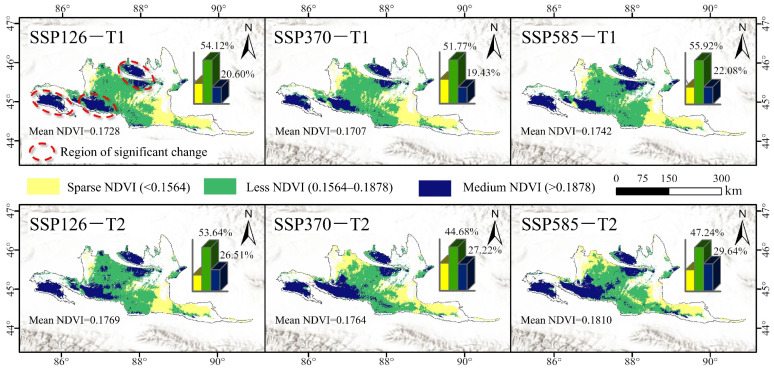
Spatial prediction of NDVI for early-spring ephemeral plants under future different climate scenarios (SSP126, SSP370 and 585).

The NDVI prediction results indicate that under future climate scenarios, the spatial distribution pattern of desert early-spring ephemeral plants is broadly similar to that of the 2001–2022 period. The Less-NDVI category remains the largest in area, while the Medium-NDVI distribution shifts northward and westward (Figure 8). During the future period 2041–2060 (T1), the NDVI distribution ranges of early-spring ephemeral plants are relatively similar across the three climate change scenarios (SSP126, SSP370, and SSP585). The Less-NDVI category accounts for over 50% of the area, whereas the Medium-NDVI category occupies 19.43–22.08%. During the future period 2061–2100 (T2), the area of the Medium-NDVI category is projected to expand, representing an increase of 24.32–40.09% compared to T1. With respect to mean NDVI, the predicted growth vigor of early-spring ephemeral plants under future scenarios follows the order SSP585 > SSP126 > SSP370, and T2 > T1. These results suggest that global warming may, to some extent, promote the proliferation of early-spring ephemeral plants.

## 3. Discussion

### 3.1. A Remote Sensing Analysis Framework for Extracting Early-Spring Ephemeral Plants in Gurbantunggut Desert

Based on the 8-day composite remote sensing data from 2001 to 2022, the NDVI time series curves in the study area showed a rapid increase, corresponding to the regreening, growth, and flourishing of early-spring ephemeral plants. The curves peaked in mid-May, after which xerophytic plants began to green up and grow gradually. Subsequently, the NDVI declined substantially to a minimum around early June, then exhibited an upward trend. Given the growth characteristics of desert plants, this pattern is consistent with the senescence of early-spring ephemeral plants and the subsequent growth of xerophytic plants. Based on this analysis, we were able to distinguish the contrasting growth characteristics of early-spring ephemeral plants and xerophytic plants. By using the 22-year mean NDVI curves and the frequency distribution of extreme values, we effectively minimized the influence of xerophytic plants in the remote sensing data.

First, the threshold range for NDVI extraction of ephemeral plants was determined based on the observed maximum and minimum values of the NDVI curves in the study area. Second, considering the concurrent flourishing of xerophytic plants, extracting the NDVI of ephemeral plants solely during the transition from peak growth to rapid decline, based on a difference index, may underestimate the early-spring ephemeral plant growth in the study area. Therefore, the 2001–2022 mean value of the difference index was used as the threshold for information extraction. Finally, using the life-cycle approach, the NDVI data corresponding to the growth and development of desert early-spring ephemeral plants were extracted by applying the segment extraction technique. Validation based on natural occurrence points also indicated that the accuracy of the extracted results was satisfactory (Table 1).

**Figure 9:**
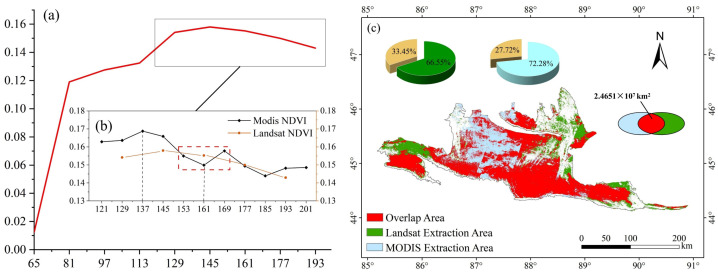
(**a**) Landsat-derived NDVI trend map. (**b**) Enlarged area comparing MODIS and Landsat NDVI time series during the ephemeral plant growth period. (**c**) Spatial comparison of ephemeral plant distribution derived from MODIS and Landsat. Different colors distinguish the two sensors.

In addition, the NDVI values extracted from the MODIS and Landsat datasets were generally consistent, with a difference in distribution area of less than 10%. Furthermore, the area of overlapping distribution was approximately 2.4651 × 10^7^ km^2^. In the core areas of early-spring ephemeral plant distribution in the study area, such as the southern margin and the distribution patches in the northern and eastern desert, the results from the MODIS and Landsat datasets showed a high degree of consistency (Figure 9c). Some discrepancies were observed in the central desert region, which may be attributed to the difference in temporal resolution between the two datasets. In comparison, the MODIS NDVI dataset, with a temporal resolution of 8 days, demonstrated a superior ability to capture key phenological changes during the growth period of early-spring ephemeral plants (Figure 9a,b). The multiple validation results confirm that this study has constructed a technically sound and logically coherent system and set of parameters for extracting information on early-spring ephemeral plants in desert environments from remote sensing data. The NDVI values of ephemeral plants extracted in this study ranged from 0.08 to 0.58, with a mean value of 0.1715, which is broadly consistent with the NDVI range reported for ephemeral plants in the arid zone of northwestern China by Wang et al. [11].

### 3.2. Spatio-Temporal Dynamics of Early-Spring Ephemeral Plants and Their Environmental Response

Over the past 22 years, the NDVI of early-spring ephemeral plants in the Gurbantunggut Desert has exhibited a long-term, slow increasing trend (Figure 2). The proportion of the Medium-NDVI area increased by over 60%, which is consistent with the findings of Duan et al. [28]. Previous studies indicate that the germination, growth, and population dynamics of early-spring ephemeral plants are strongly dependent on regional hydrothermal conditions and their configuration [19,29]. Consequently, these plants typically display distinct selective distribution patterns and pronounced eco-geographical patterns [17]. During this 22-year period, early-spring ephemeral plants in the Gurbantunggut Desert were primarily concentrated in the oasis–desert transition zone along the southern margin of the desert (Figure 3). These areas are predominantly characterized by fixed and semi-fixed dunes and sandy lands. Precipitation in April and May is particularly significant, accounting for more than 29.0% of the desert’s total annual precipitation [30,31]. This “spring-rain-dominated” precipitation pattern has considerable eco-geographical significance, as it improves moisture conditions within the sand layer and enhances spring soil moisture.

As the only desert in China with stable winter snow cover, the Gurbantunggut Desert experiences snow depths that can exceed 20 cm [32]. Along the southern desert margin, the number of snow days remains stable at around 80 days. With rising temperatures, snowmelt runoff in this southern region is substantially higher than in the eastern and western parts, providing highly favorable moisture conditions for the development of these plants (Supplementary Materials, Figure S2). Consequently, the southern margin of the Gurbantunggut Desert constitutes a crucial ecological habitat for the survival, proliferation, and succession of early-spring ephemeral plants. At the same time, the luxuriant growth and diverse distribution patterns of these plants play a key role in stabilizing this desert ecosystem [28]. However, the growth and development of early-spring ephemeral plants are highly sensitive to changes in climatic conditions, resulting in dynamic and opportunistic characteristics in their spatial distribution [5]. Even small-scale precipitation events (≤5 mm) can significantly influence their growth and reproductive strategies [33]. This sensitivity partly accounts for the oscillatory fluctuations and northward migration trend observed in the distribution centroids of early-spring ephemeral plants across the different growth vigor classes (Figure 5d–f).

Regarding driving mechanisms, this study found that the growth and development of early-spring ephemeral plants during early spring primarily depend on snow and precipitation factors. These two factors together contribute over 60% of the influence (Figure 6), and the dominant role of precipitation shows an increasing trend (Figure 7e). Related studies also indicate that species number of desert ephemeral plants increase with increasing precipitation [34]. During 2016–2022, early-spring ephemeral plants exhibited above-average growth, yet a progressive decline in NDVI was observed across successive years (Figure 2). This trend coincided with reductions in precipitation: annual precipitation, precipitation of the driest quarter, and March–June precipitation all decreased significantly (Supplementary Materials, Figure S3). These findings underscore the overriding influence of water availability on desert vegetation productivity. Although elevated temperatures may indirectly constrain seed germination and early growth of ephemeral plants by accelerating snowmelt, intensifying evaporation, and causing earlier cessation of snow cover (Supplementary Materials, Figure S3), the magnitude of these negative effects has progressively diminished (Figure 7e). The overall effect of the topographic factor is negative, as corroborated by the observed migration of the distribution centroid of these plants toward lower elevations over the 22-year period (Figure 5 and Figure 7). Furthermore, ephemeral plants often exhibit selective distribution patterns, favoring the mid-to-lower sections of windward slopes and the lower sections of leeward slopes on semi-fixed and semi-mobile longitudinal dunes [35,36]. This pattern reflects the unique ecological adaptation of ephemeral plants to local desert microenvironments and provides important insight into their dynamic fluctuations along the east–west axis of the desert.

Under future climate scenarios, the southern margin of the Gurbantunggut Desert is projected to remain a highly suitable hotspot for the growth and distribution of early-spring ephemeral plants, albeit with a westward migration trend [37] (Figure 8). This region offers relatively favorable hydrothermal conditions and pronounced topographic relief, featuring sand ridges 10–30 m high, barchan dune chains, and honeycomb dunes. These landforms provide optimal microhabitats and dispersal corridors for the growth, local adaptation, and migration of ephemeral plants [38,39]. Furthermore, previous studies have shown that the widely distributed Salix shrubs along the southern desert margin can facilitate the growth of early-spring ephemeral plants by reducing wind speed and improving microclimate conditions [11]. Meanwhile, the high-altitude mountains and canyons in the southern part of the desert also serve as natural refugia, enabling these plants to cope with climate change [40].

Moreover, early-spring ephemeral plants are projected to establish a second major habitat for survival and reproduction in the northern part of the desert, distinct from the southern margin (Figure 8). This finding is consistent with the characteristic pattern observed in arid zones globally, where plant species distributions shift toward higher latitudes and altitudes under global warming [41]. Specifically, the warming and wetting trend in Xinjiang is projected to persist until the end of the 21st century under various future climate scenarios [42]. This increased warmth and moisture may benefit the growth and proliferation of these plants. During 2081–2100 (T2), compared to 2041–2060 (T1), the Medium-NDVI area within the Gurbantunggut Desert is expected to expand, with an increase ranging from 24.32% to 40.09%.

### 3.3. Conservation Prioritization: Insights and a Framework for Ephemeral Plant Diversity

Long-term survival and evolution under the extreme environmental conditions of the Gurbantunggut Desert have endowed early-spring ephemeral plants with strong ecological adaptability to arid and sandy environments, making them distinctive plant resources of this desert [43]. In terms of their productive value, early-spring ephemeral plants serve as excellent spring forage in the seasonal rotational grazing systems of northern Xinjiang, including species such as *Bromus tectorum* L. and *Adonis amurensis*. A few species, such as *Fritillaria cirrhosa* and *Ferula teterrima*, are also used as traditional Chinese medicinal materials. However, many early-spring ephemeral plant species are now facing the threat of extinction, including *Ferula dubjanskyi* and *Eremosparton songoricum*. Therefore, it is urgent to strengthen the conservation of the species diversity of early-spring ephemeral plants.

This study identified the southern edge of the Gurbantunggut Desert as the core area of concentrated distribution of early-spring ephemeral plants with Medium-NDVI values (Figure 3); this region is also a hotspot for ephemeral plant species diversity [28]. Under future warmer and wetter climate conditions, both the extent and the NDVI values of the Medium-NDVI category are projected to expand significantly; nevertheless, the southern desert edge will remain a central area for the vigorous growth of ephemeral plants. As the only cold desert in the world with stable winter snow cover, the Gurbantunggut Desert sustains a unique concentration of early-spring ephemeral plants. Therefore, priority protection should be given to this region of high biodiversity importance. Moreover, under extreme climatic conditions, desert edges often function as refugia and ecological migration corridors for the survival and expansion of desert plant species [19]. Thus, establishing small-scale nature reserves to effectively protect the diversity of early-spring ephemeral plants at the southern edge of the desert holds critical ecological and biogeographical significance for the overall diversity and continued succession of these species.

Our results further indicate that under the three climate scenarios, a second highly suitable distribution area for early-spring ephemeral plants may emerge in the northern part of the desert (Figure 8). However, given the complexity and uncertainty inherent in species migration and dispersal processes [44], constructing ecological corridors with lower ecological resistance in desert regions would facilitate the migration of ephemeral plant species and vegetation succession, thereby contributing to the conservation and enhancement of biodiversity.

### 3.4. Limitations and Outlook

Although this study integrated NDVI time series, random forest (RF), structural equation modeling (SEM), and convolutional neural network (CNN) approaches to systematically investigate the spatiotemporal distribution patterns, environmental drivers, and future dynamics of early-spring ephemeral plants in the Gurbantunggut Desert, several limitations remain. First, vegetation identification and dynamic monitoring were primarily based on MODIS NDVI time series data, whose spatial resolution was resampled to 1 km. In desert ecosystems, this may introduce a mixed-pixel problem. Because ephemeral plants are typically distributed in patches, the 1 km spatial resolution may not fully capture their actual distribution range, thereby affecting the identification accuracy to some extent. Second, the multi-source data fusion process involved datasets with different spatial resolutions. Although a series of methods were applied to harmonize the scales of different data sources, the scale transformation may still lead to the loss of local spatial information, which in turn influences the modeling results. Furthermore, global climate models (GCMs) are mathematical abstractions of the complex climate system, and differences in the understanding and parameterization schemes of key climate processes among models lead to divergent climate response simulations under the same emission scenarios. The BCC-CSM2-MR model adopted in this study performs well overall in the China region [45]; however, a single model cannot fully reflect the internal variability of the climate system or the range of uncertainty across multiple models, potentially introducing biases in the estimated magnitude of future climate change [46,47]. Therefore, the projected results remain subject to a certain degree of uncertainty.

To address the above limitations, future research can be deepened in several aspects. On the one hand, Sentinel-2 imagery, GaoFen series satellite data, or even unmanned aerial vehicle (UAV) hyperspectral data can be introduced, and spectral unmixing techniques can be applied to separate endmembers within pixels, so as to overcome the limitation of MODIS mixed pixels and achieve a more accurate delineation of early-spring ephemeral plant distribution. On the other hand, dynamic models of snow cover and topography should be developed to replace the current assumption of static environmental variables, and multiple GCMs can be incorporated to provide more realistic projections of suitable habitats for early-spring ephemeral plants under different climate scenarios. In addition, establishing long-term monitoring plots in the core distribution area along the southern margin of the Gurbantunggut Desert and in potential suitable areas in the north, and synchronously collecting population dynamics, micrometeorological, and soil moisture observation data, will allow the empirical linking of autecological processes with remotely sensed community signals. Ultimately, this will enable a comprehensive understanding from macro-scale patterns to micro-scale mechanisms, providing more robust and actionable scientific evidence for biodiversity conservation in arid regions.

## 4. Materials and Methods

### 4.1. Study Area

**Figure 10:**
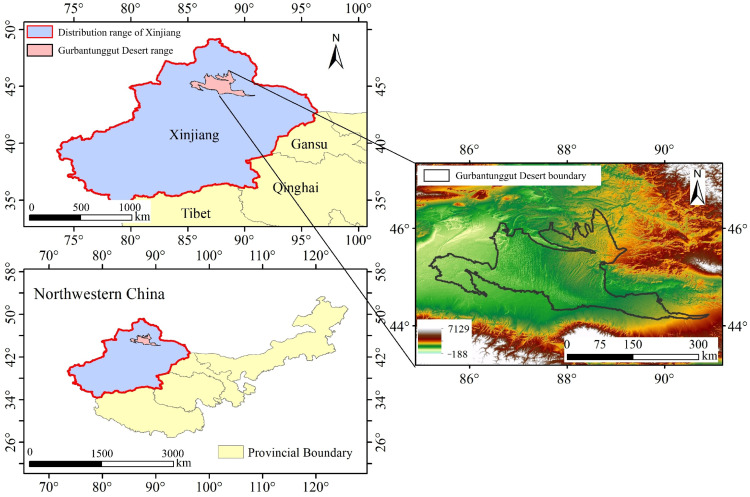
Location of the study area.

The Gurbantunggut Desert, located in the Junggar Basin of northern Xinjiang, China, is the largest fixed and semi-fixed desert in China and represents a typical cold desert ecosystem in Central Asia (Figure 10). The region has a continental arid climate with hot, dry summers and cold winters. Mean annual precipitation is relatively low, yet more than 40% of the annual total falls in winter and early spring, mostly as snow. Stable snow cover generally persists for 70–130 days each winter, providing crucial moisture for the growth of early-spring ephemeral plants through snowmelt infiltration. The desert landscape is dominated by fixed and semi-fixed dunes, longitudinal sand ridges, and interdune lowlands, which together create diverse microhabitats. Owing to the distinctive hydrothermal conditions and stable winter snow cover, the Gurbantunggut Desert harbors abundant early-spring ephemeral plants, particularly along the southern desert margin and the oasis–desert ecotone. These plants complete their life cycle rapidly from early spring to early summer and play a critical ecological role in stabilizing sandy surfaces, maintaining desert biodiversity, and sustaining ecosystem functioning.

### 4.2. Materials

#### 4.2.1. NDVI Data

Surface reflectance data from MODIS and Landsat 8 were acquired through the Google Earth Engine (GEE) platform (https://code.earthengine.google.com/). This study used the MOD09A1 surface reflectance product (8-day composite, 500 m resolution) provided by the National Aeronautics and Space Administration (NASA). To capture growth variations of early-spring ephemeral plants during their critical growth period, we extracted data spanning March to July of each year from 2001 to 2022. Concurrently, Landsat 8 Operational Land Imager (OLI) data (16-day revisit cycle, 30 m resolution) from the U.S. Geological Survey (USGS) for 2016–2020 were employed to further validate the NDVI information of early-spring ephemeral plants extracted from MOD09A1 data in the study area. All preprocessing procedures—including cloud removal, vegetation index calculation, clipping, and reprojection—were performed within the GEE platform.

#### 4.2.2. Environmental Data

**Figure 11:**
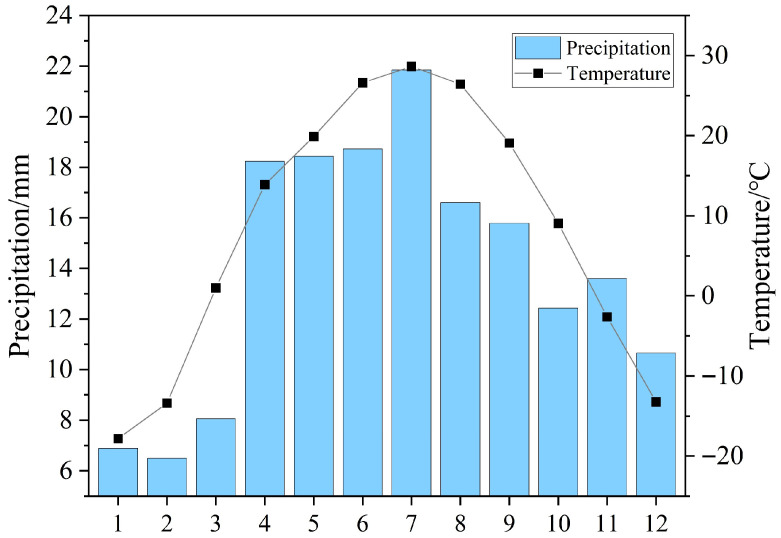
Trends in monthly mean Temperature and Precipitation across the Gurbantunggut Desert (2001–2022).

The environmental variables selected in this study include four categories: temperature, precipitation, snow, and topography; detailed descriptions are provided in Table 4. Based on the observed monthly temperature and precipitation variations from 2001 to 2022 (Figure 11), we identified the driest quarter (January–March), the wettest quarter (May–July), the warmest quarter (June–August), and the coldest quarter (December–February of the following year) in the study area. In addition, according to the phenological cycle of early-spring ephemeral plants, their growing season was defined as March to June.

Future climate data were obtained from the downscaled CMIP6 projections released by WorldClim v2.1. The BCC-CSM2-MR global climate model, which performs well over China, was selected, and three typical shared socioeconomic pathway (SSP) scenarios were adopted: SSP1-2.6 (low emissions), SSP3-7.0 (medium-to-high emissions), and SSP5-8.5 (high emissions). The potential impacts of climate change on early-spring ephemeral plants in the Gurbantunggut Desert were analyzed for two future periods: 2041–2060 (T1) and 2081–2100 (T2). The BCC-CSM2-MR model has been shown to achieve spatial correlation coefficients of 0.96 for temperature and 0.73 for precipitation over China, indicating high reliability [27].

To minimize errors arising from scale transformation, all datasets underwent radiometric calibration, atmospheric correction, and cloud masking. For raster grids with missing values, the inverse distance weighting (IDW) method was applied in MATLAB (R2023a) to achieve smooth interpolation and ensure spatial continuity. Subsequently, all data were resampled to a unified output extent, cell size (1 km × 1 km), and projection parameters using the nearest neighbor method, ensuring complete consistency in the resampling process across different data sources. Importantly, each dataset was resampled to the target resolution only once to avoid error accumulation caused by repeated resampling.

#### 4.2.3. Species Occurrence Data

Species occurrence data were obtained from multiple biodiversity databases, including the China Virtual Herbarium (CVH, http://www.cvh.org.cn (accessed on 9 April 2025)), the National Specimen Information Infrastructure (NSII, http://www.nsii.org.cn (accessed on 9 April 2025)), the Global Biodiversity Information Facility (GBIF, https://www.gbif.org/zh/ (accessed on 9 April 2025)), and iPlant (http://www.iplant.cn/). We selected 30 representative and ecologically important early-spring ephemeral plant species with documented geographical distributions in the Gurbantunggut Desert. To ensure the reliability of the model simulations, we excluded species with fewer than five valid occurrence points [48]. Spatial deviations in the distribution records were corrected using Google Earth, and duplicate records within a 0.1 km grid were removed using R (Version 4.3.1). Ultimately, 185 valid occurrence points were retained.

### 4.3. Methods

#### 4.3.1. NDVI Remote Sensing Identification and Extraction of Ephemeral Plants

NDVI was calculated from the MOD09A1 surface reflectance product using the standard formula:

(1) $$NDVI=\frac{\rho_{NIR}-\rho_{RED}}{\rho_{NIR}+\rho_{RED}}$$

where $\rho_{NIR}$ and $\rho_{RED}$ denote the near-infrared and red band surface reflectance, respectively.

To eliminate interference from the surrounding oasis–desert transitional zone, the largest rectangular area within the central desert was selected as the statistical region for constructing the NDVI time series curves. This region spans approximately 218.13 km from east to west and 135.63 km from north to south. For each pixel, an 8-day composite NDVI time series was extracted from Day of Year (DOY) 65 to 225 of each year, a time window that fully covers the entire life cycle of early-spring ephemeral plants—from regreening to complete senescence.

**Figure 12:**
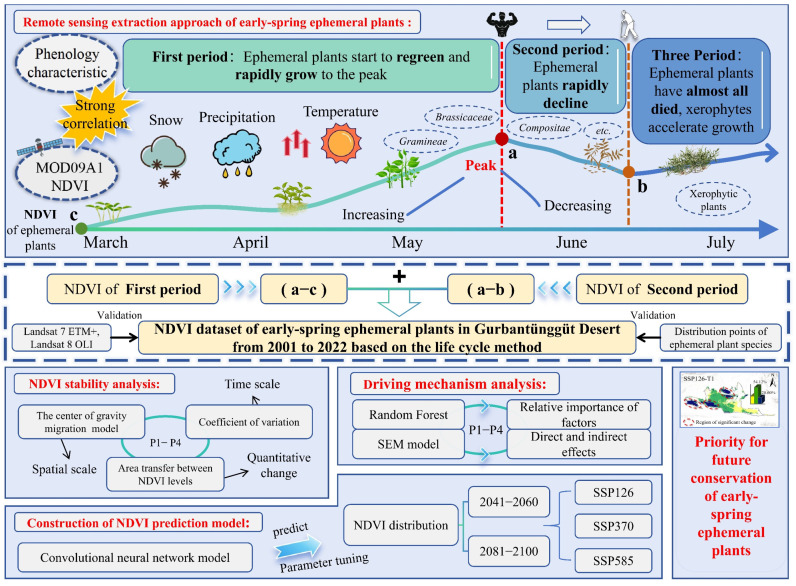
Methodological framework of this study.

Based on the NDVI time series, three key phenological feature points were extracted: the regreening onset point ($DOY_{C}$), the peak growth point ($DOY_{a}$), and the post-peak minimum point during the rapid decline stage ($DOY_{b}$). These key points characterize the typical “rapid growth–rapid decline” life cycle of ephemeral plants (Figure 12). Leveraging the life-cycle variation characteristics, the NDVI contribution of ephemeral plants in a given year is defined as the sum of two NDVI increments:

(2) $$NDVI_{eph}=\left(NDVI_{a}-NDVI_{c}\right)+\left(NDVI_{a}-NDVI_{b}\right)$$

where $NDVI_{a}-NDVI_{c}$ represents the NDVI increase from the regreening onset to the peak growth stage, reflecting the rapid vegetation growth process, and $NDVI_{a}-NDVI_{b}$ represents the NDVI decrease from the peak to the rapid decline stage, capturing the fast senescence of ephemeral plants. Together, these two components quantify the dynamic intensity of ephemeral plants throughout their entire growth cycle.

To reduce the interference of secondary growth of xerophytic shrubs in summer on the NDVI signal, the 2001–2022 multi-year mean value was used as a threshold to re-extract the NDVI increment during the rapid decline stage of ephemeral plants. Regions exceeding this mean value were identified as the distribution area of ephemeral plants, and the accuracy was subsequently validated using actual species occurrence data.

Based on the natural breaks method, the NDVI contribution of early-spring ephemeral plants from 2001 to 2022 in the study area was classified into three categories: Medium-NDVI (NDVI > 0.1878), Less-NDVI (0.1564 < NDVI < 0.1878), and Sparse-NDVI (0 < NDVI < 0.1564).

#### 4.3.2. Spatio-Temporal Characteristics Analysis

This study applied Ensemble Empirical Mode Decomposition (EEMD) to analyze the interannual NDVI time series of early-spring ephemeral plants (Figure 12). Through a sifting process, EEMD adaptively decomposes the original signal into a finite set of intrinsic mode functions (IMFs) of different frequencies and a residual trend component. The variance contribution rate of each IMF was calculated to quantify its relative contribution to the overall variability of the original NDVI signal [49,50]. This approach allows robust detection of periodic oscillations and long-term trends in non-stationary and non-linear time series [40].

Piecewise linear regression was employed to identify temporal breakpoints in the NDVI trajectory [51,52]. In addition, the centroid shift model and the coefficient of variation (CV) were used to quantify the directional migration of the NDVI centroid and the spatial stability of early-spring ephemeral plant distribution, respectively [53,54] (Figure 12).

#### 4.3.3. Analysis of Environmental Drivers and Their Mechanisms

Taking advantage of the nonlinear fitting capability and ensemble learning characteristics of the random forest (RF) model, this study quantified the relative contributions of environmental drivers to the NDVI of early-spring ephemeral plants using Gini importance [24]. Pearson correlation coefficients were calculated to identify associations between environmental drivers and NDVI. Beyond their direct effects, environmental drivers may also indirectly influence the growth of ephemeral plants through interactions with other factors. To examine these direct and indirect pathways, a structural equation model (SEM) based on partial least squares (PLS-SEM) was developed [55,56]. The SEM was implemented using the plspm package in R, and model performance was evaluated using the goodness-of-fit (GoF) index.

#### 4.3.4. Development and Validation of the Ephemeral Plant NDVI Model

**Figure 13:**
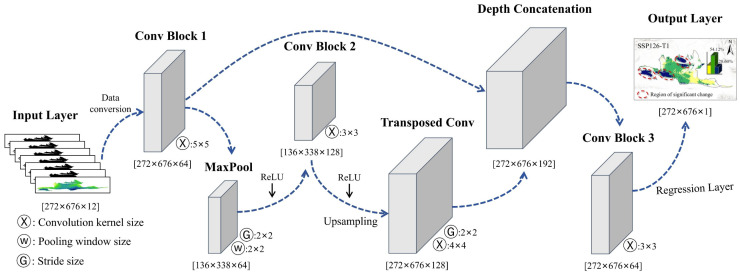
Schematic of the proposed convolutional neural network (CNN) architecture of this study.

A convolutional neural network (CNN) model was used to predict the spatial distribution patterns of early-spring ephemeral plant NDVI under future climate change scenarios. Given the high sensitivity of ephemeral plants to environmental change and their fine-scale spatial distribution characteristics, CNN models are particularly effective at extracting hierarchical spatial features. These models can automatically learn and capture relevant patterns and relationships embedded in the spatial structure of the data, thereby effectively identifying subtle variations. To reduce the risk of overfitting, five-fold cross-validation was incorporated during model construction [24,25]. The model architecture is illustrated in Figure 13. Root mean square error (RMSE) and the coefficient of determination (R^2^) were selected as evaluation metrics to validate the predictive accuracy of the model [57,58].

## 5. Conclusions

This study developed an integrated remote sensing framework that combines NDVI time-series phenological features, machine learning, and multi-source environmental variables to systematically reveal the spatiotemporal dynamics of early-spring ephemeral plants and the underlying climate-driven mechanisms in the Gurbantunggut Desert. The results show that: (1) the life-cycle-based NDVI extraction method achieved high classification accuracy (>80%). From 2001 to 2022, the distribution of early-spring ephemeral plants exhibited an overall increasing trend, with a spatial pattern characterized by northward expansion from the southern desert margin and a continuous northward shift of the NDVI centroid. (2) Snow-related processes (snowend, snowday) and precipitation in the driest quarter were identified as the dominant drivers controlling plant growth and distribution, jointly explaining more than 60% of the spatial variability, while temperature and topographic factors exerted indirect influences. (3) Under future climate scenarios, Medium-NDVI areas are projected to expand continuously, with suitable habitats shifting northward and westward, suggesting that climate warming may substantially promote the spatial reorganization and latitudinal migration of early-spring ephemeral plants. Overall, this study highlights the critical role of winter snow processes and spring water availability in shaping the distribution patterns of early-spring ephemeral plants in cold arid deserts, and reveals their potential expansion pathways and response mechanisms under climate change. These findings provide new insights into vegetation adaptation in arid regions and offer scientific support for biodiversity conservation and ecological corridor planning under a changing climate.

## Figures and Tables

**Table 1:** Accuracy assessment of early-spring ephemeral plants classification in the Gurbantunggut Desert (2001–2022).

Species Name	Number of Validation Points	Points Within Range	Points Outside Range	Accuracy
Sum	185	152	33	82.16%
*Brassicaceae*	50	48	2	96.00%
*Compositae*	29	25	4	86.20%
*Gramineae*	26	21	5	80.77%
*Boraginaceae*	14	12	2	85.71%
*Liliaceae*	13	8	5	61.54%
*Umbelliferae*	11	8	3	72.73%
*Geraniaceae*	19	14	5	73.68%
*Chenopodiaceae*	11	10	1	90.91%
*Leguminosae*	7	2	5	28.57%
*Plantaninaceae*	5	1	4	20.00%
*Papaveraceae*	5	3	2	60.00%

Note: “Points Within Range” indicates the actual species occurrence points that fall within the distribution mask of early-spring ephemeral plants extracted in this study.

**Table 2:** Period and variance contribution rate of intrinsic mode function (IMF) components.

	IMF1	IMF2	IMF3	Residual (Res)
Period	1.4 a	3.0 a	6.5 a	-
Variance Contribution Rate	67.22%	24.78%	2.12%	5.89%

Note: “a” represents 1 year.

**Table 3:** Transition area of NDVI distribution for early-spring ephemeral plants in the Gurbantunggut Desert (2001–2022).

Transfer Type		Transfer Area Between Time Periods (km^2^)		
		2001–2007 to 2008–2012	2008–2012 to 2013–2015	2013–2015 to 2016–2022
Sparse	Sparse	7669	10,934.25	4167.25
Sparse	Less	1261.5	1050.25	10,006.25
Sparse	Medium	46.5	3.5	2159.5
Less	Sparse	4049.75	5231.5	37
Less	Less	12,025.75	11,367	7531.5
Less	Medium	1668.5	223.75	8020.5
Medium	Sparse	269.25	167.25	2
Medium	Less	3535	3171.75	672.5
Medium	Medium	4157.75	2533.75	2691.75

**Table 4:** List and description of the environmental variables.

Variable Category	Variable	Year	Resolution	Description and Units	Data Sources
Temperature factors	Temp_year	2001–2022	1 km	Annual mean temperature of historical years (°C)	National Tibetan Plateau Data Center (TPDC) (https://data.tpdc.ac.cn)
	Temp_AU			Mean Temperature of driest quarter of historical years (°C)	
	Temp_SP			Mean Temperature of warmest quarter of historical years (°C)	
	Temp_GS			Mean temperature of from March to June (°C)	
Precipitation factors	Pre_year	2001–2022	1 km	Annual Precipitation of historical years (mm)	National Tibetan Plateau Data Center (TPDC) (https://data.tpdc.ac.cn)
	Pre_AU			Precipitation of driest quarter of historical years (mm)	
	Pre_SP			Precipitation of warmest quarter of historical years (mm)	
	Pre_GS			Mean precipitation in from March to June (mm)	
Snow factors	Snowmelt	2001–2022	500 m	Snowmelt water (mm)	National Cryosphere Desert Data Center (NCDC) (http://www.ncdc.ac.cn)
	Snowday			Number of snow days (day)	
	Snowstart			Start day of snow (day)	
	Snowend			End day of snow (day)	
Topographic factors	Elevation	-	90 m	Altitude of study area (m)	Geospatial Data Cloud (GDC) (http://www.gscloud.cn)
	Slope			Slope of study area (°)	
	Aspect			Aspect of study area	
Future climate data	Bio1	2041–2060 2061–2080	30 s	Annual mean temperature (°C)	WorldClim v2.1 database (https://www.worldclim.org)
	Bio9			Mean temperature of Driest Quarter (°C)	
	Bio10			Mean temperature of Warmest Quarter (°C)	
	Bio12			Annual Precipitation(mm)	
	Bio17			Precipitation of Driest Quarter (mm)	
	Bio18			Precipitation of Warmest Quarter (mm)	

## Data Availability

Data will be made available on request.

## Supplementary Materials

The following supporting information can be downloaded at: https://www.mdpi.com/article/10.3390/plants15101586/s1, Figure S1: Statistical correlations of climatic and topographic factors with NDVI in early-spring ephemeral plants in the Gurbantunggut Desert during periods P1 (a), P2 (b), P3 (c), and P4 (d). Figure S2: Spatial distribution of key climate drivers in the Gurbantunggut Desert (2001–2022). Figure S3: Temporal variation characteristics of key climate drivers in the Gurbantunggut Desert (2001–2022).
