# Supplementary material for: Climate-Driven Redistribution of Early-Spring Ephemeral Plant Communities in Cold Arid Deserts: Evidence from the Gurbantunggut Desert, China
Source: Plants (Basel). 2026 May 21;15(10):1586. doi: 10.3390/plants15101586 (PMC13211235; doi:10.3390/plants15101586)
Supplement: Supplementary file 1 [file plants-15-01586-s001.zip › plants-4278644-supplementary.docx]

**Figure S1.** Statistical correlations of climatic and topographic factors with NDVI in early-spring ephemeral plants in the Gurbantunggut Desert during periods P1(a), P2(b), P3(c), and P4(d).


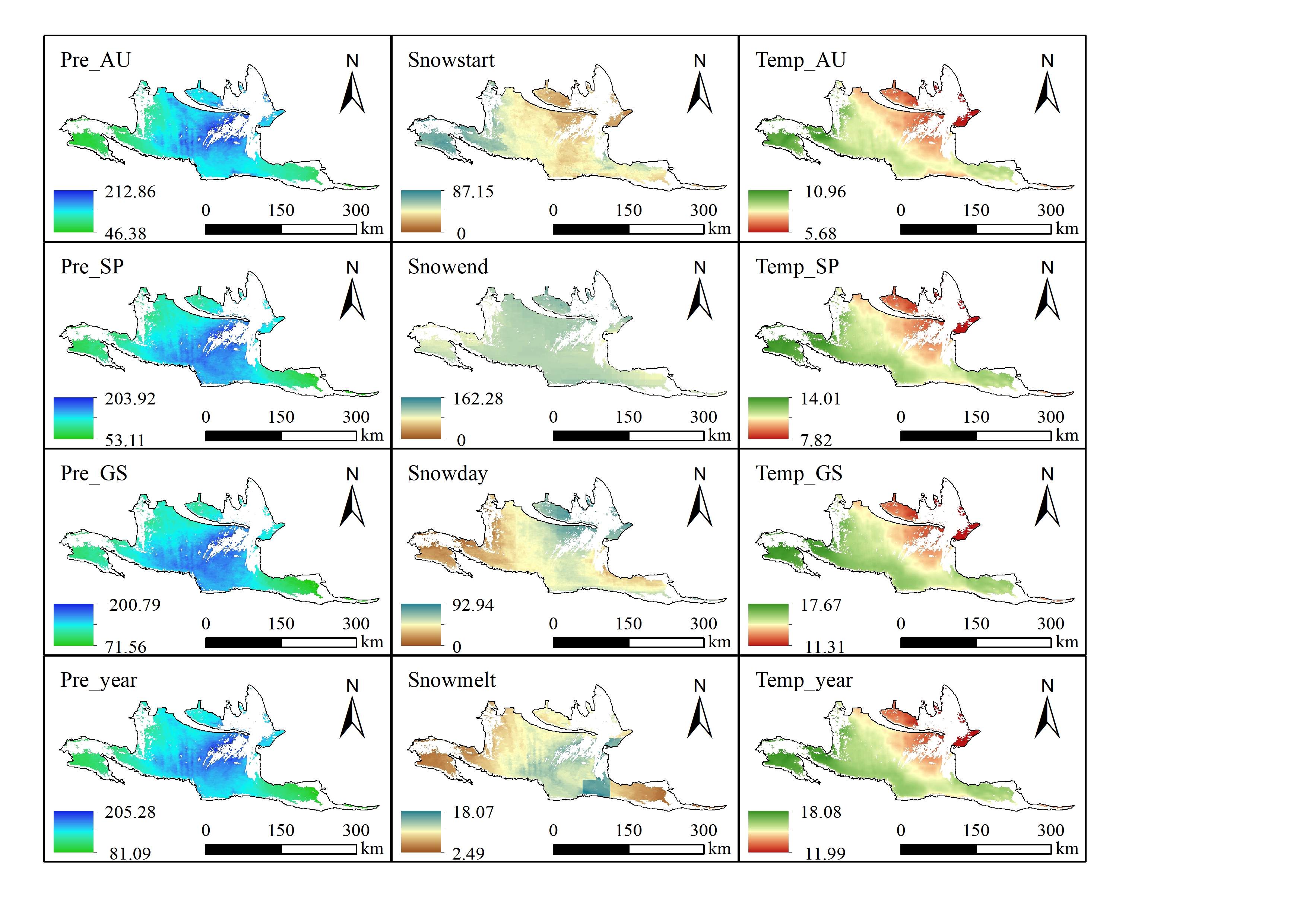


**Figure S2.** Spatial distribution of key climate drivers in the Gurbantunggut Desert (2001-2022).


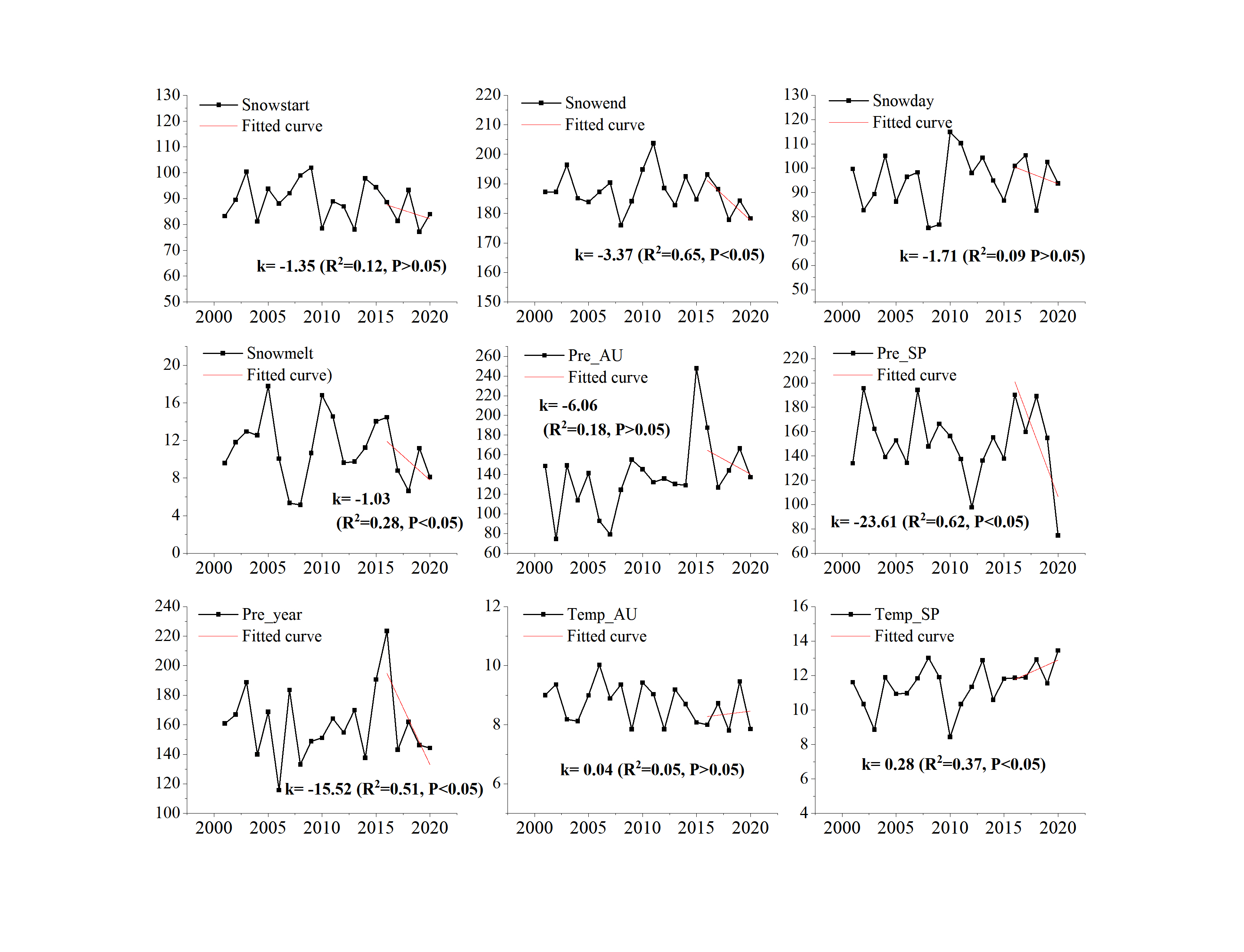


**Figure S3.** Temporal variation characteristics of key climate drivers in the Gurbantunggut Desert (2001-2022).
